# Supplementary material for: Causal Relationship between Aging and Anorexia Nervosa: A White-Matter-Microstructure-Mediated Mendelian Randomization Analysis
Source: Biomedicines. 2024 Aug 16;12(8):1874. doi: 10.3390/biomedicines12081874 (PMC11351342; doi:10.3390/biomedicines12081874)
Supplement: Supplementary file 1 [file biomedicines-12-01874-s001.zip › Supplementary Figure/Figure S4/Singe SNP plot of MR analysis for TL to ubm-b-1958.pdf]

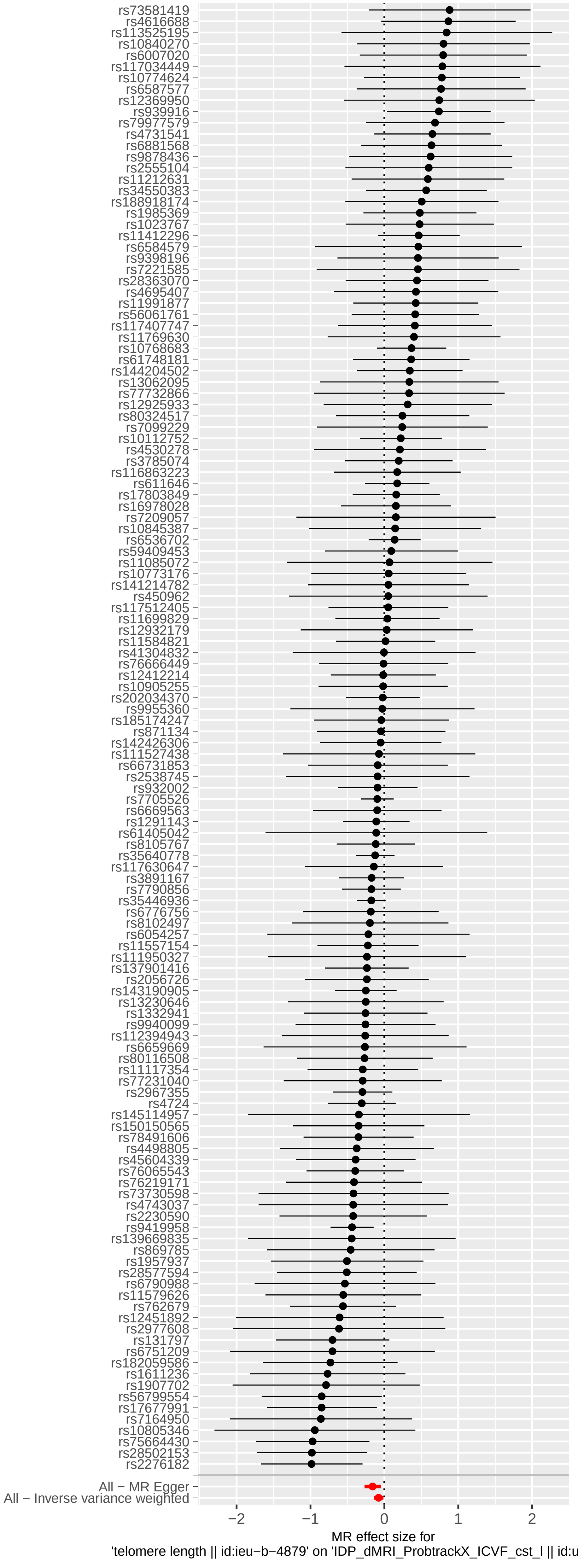
rs73581419
rs4616688
rs113525195
rs10840270
rs6007020
rs117034449
rs10774624
rs6587577
rs12369950
rs939916
rs79977579
rs4731541
rs6881568
rs9878436
rs2555104
rs11212631
rs34550383
rs188918174
rs1985369
rs1023767
rs11412296
rs6584579
rs9398196
rs7221585
rs28363070
rs4695407
rs11991877
rs56061761
rs117407747
rs11769630
rs10768683
rs61748181
rs144204502
rs13062095
rs77732866
rs12925933
rs80324517
rs7099229
rs10112752
rs4530278
rs3785074
rs116863223
rs611646
rs17803849
rs16978028
rs7209057
rs10845387
rs6536702
rs59409453
rs11085072
rs10773176
rs141214782
rs450962
rs117512405
rs11699829
rs12932179
rs11584821
rs41304832
rs76666449
rs12412214
rs10905255
rs202034370
rs9955360
rs185174247
rs871134
rs142426306
rs111527438
rs66731853
rs2538745
rs932002
rs7705526
rs6669563
rs1291143
rs61405042
rs8105767
rs35640778
rs117630647
rs3891167
rs7790856
rs35446936
rs6776756
rs8102497
rs6054257
rs11557154
rs111950327
rs137901416
rs2056726
rs143190905
rs13230646
rs1332941
rs9940099
rs112394943
rs6659669
rs80116508
rs11117354
rs77231040
rs2967355
rs4724
rs145114957
rs150150565
rs78491606
rs4498805
rs45604339
rs76065543
rs76219171
rs73730598
rs4743037
rs2230590
rs9419958
rs139669835
rs869785
rs1957937
rs28577594
rs6790988
rs11579626
rs762679
rs12451892
rs2977608
rs131797
rs6751209
rs182059586
rs1611236
rs1907702
rs56799554
rs17677991
rs7164950
rs10805346
rs75664430
rs28502153
rs2276182
All − MR Egger
All − Inverse variance weighted
−2
−1
0
1
2
MR effect size for
'telomere length || id:ieu−b−4879' on 'IDP_dMRI_ProbtrackX_ICVF_cst_l || id:u
